# How to characterize the public health workforce based on essential public health operations? environmental public health workers in the Netherlands as an example

**DOI:** 10.1186/s12889-015-2095-5

**Published:** 2015-08-06

**Authors:** M. Jambroes, R. van Honschooten, J. Doosje, K. Stronks, M. L. Essink-Bot

**Affiliations:** Department of Public Health, J2-216, Academic Medical Center/University of Amsterdam, PO Box 22660, 1100 DD Amsterdam, The Netherlands; National Association of Public Health Services (GGDGHOR-Nederland),, Zwarte Woud 2, 3524 SJ Utrecht, The Netherlands

**Keywords:** Public health workforce, Essential public health operations, Workforce enumeration, Workforce planning

## Abstract

**Background:**

Public health workforce planning and policy development require adequate data on the public health workforce and the services provided. If existing data sources do not contain the necessary information, or apply to part of the workforce only, primary data collection is required. The aim of this study was to develop a strategy to enumerate and characterize the public health workforce and the provision of essential public health operations (EPHOs), and apply this to the environmental public health workforce in the Netherlands as an example.

**Methods:**

We specified WHO’s EPHOs for environmental public health and developed an online questionnaire to assess individual involvement in these. Recruitment was a two-layered process. Through organisations with potential involvement in environmental public health, we invited environmental public health workers (n = 472) to participate in a national survey. Existing benchmark data and a group of national environmental public health experts provided opportunities for partial validity checks.

**Results:**

The questionnaire was well accepted and available benchmark data on physicians supported the results of this study regarding the medical part of the workforce. Experts on environmental public health recognized the present results on the provision of EPHOs as a reasonable reflection of the actual situation in practice.

All EPHOs were provided by an experienced, highly educated and multidisciplinary workforce. 27 % of the total full-time equivalents (FTEs) was spent on EPHO ‘assuring governance for health’. Only 4 % was spent on ‘health protection’. The total FTEs were estimated as 0.66 /100,000 inhabitants.

**Conclusions:**

Characterisation of the public health workforce is feasible by identification of relevant organisations and individual workers on the basis of EPHOs, and obtaining information from those individuals by questionnaire. Critical factors include the operationalization of the EPHOS into the field of study, the selection and recruitment of eligible organisations and the response rate within organisations.. When existing professional registries are incomplete or do not exist, this strategy may provide a start to enumerate the quantity and quality of the public health within or across countries.

## Background

Recently, the review of the public health capacity in Europe in 2013 by the European Commission Directorate General for Health and Consumers showed uncertainty regarding the capacity of the public health workforce in Europe [1]. Adequate data on the size and composition of the actual workforce are needed to support workforce planning and policy development, in order to guarantee sufficient and competent workers in the future [2–4]. Measuring the public health capacity is hence an important but challenging task.

Strategies to enumerate the public health workforce have been subject of scientific debate for many years [5–10]. Efforts to develop information about the public health workforce encountered major obstacles, including uncertain boundaries of the field of public health, and the multidisciplinary workforce in combination with the absence of credential requirements for most of the disciplines involved [1, 11, 12].

Until now, most efforts to enumerate the public health workforce are based on existing data sources, see for example the USA centers of excellence in public health workforce studies focusing on the governmental public health workforce, and the recently published study on characterization of the federal workforce at the Centers of Disease Control and Prevention [13–16].

However, the limitations of using existing databases for public health workforce enumeration are known and have been emphasised [1, 17, 18]. For instance, disparate job titles of public health professionals are a drawback, because not all job titles are accurately labelled as ‘public health’ in the different data sources [19]. Also, registrations use different definitions of public health workers, different disciplines are often registered in different registries and not all disciplines or workplace settings are represented in the databases.

If existing data sources do not contain the necessary information, or apply to part of the workforce only, primary data collection is essential for accurate characterization of the workforce. To date, no standard strategy for this primary data collection exists.

Therefore we developed a strategy aiming to 1) assess the size and composition of the multidisciplinary public health workforce across different organisations, and 2) to assess the services provided. We applied our strategy to the environmental public health workforce in the Netherlands as an example.

Environmental public health focuses on the interactions with and effects of the environment on health, e.g. indoor and outdoor pollution and chemical safety. Environmental public health is a relatively small discipline within the public health working field in the Netherlands and is mainly but not exclusively performed through local public health services (see Table 1) by a very multi-disciplinary workforce, among which physicians. The total size and composition of this workforce is unknown. However, there is a compulsory registry for physicians and the total number of environmental public health workers working at local public health services is known. Additional data relevant for workforce planning about age, educational background, job function and provision of services are not available.

**Table 1:** National Association of Local Public Health Services

‘GGDGHOR Nederland’ is the national Association of Local Public Health Services (‘GGD’en’) and GHOR(Regional Medical Emergency Preparedness and Planning) offices in the Netherlands. By law, all Dutch municipalities have the obligation to protect, control and promote the health of their inhabitants. Each municipality is associated with a local public health service to carry out these tasks. There are about 400 municipalities in the Netherlands and they are served by 26 local public health services. This means that one local public health service is often jointly directed by several municipalities. Local public health services are responsible for preventive health care. All local public health services have a number of uniform tasks, as specified in the law: the Public Health Act. Examples of those tasks are Youth health, Infectious disease control, Health promotion and Environmental public health. Environmental public health focuses on the interactions with and effects of the environment on health, e.g. indoor and outdoor pollution and chemical safety.	

The aim of the present study was to examine the feasibility and validity of our newly developed strategy to enumerate the public health workforce and the services provided, by applying it to environmental public health workforce as an example.

## Methods

### General study design

A national cross-sectional survey was conducted using an online questionnaire.

To assess the services provided by the workforce we used the recently defined essential public health operations (EPHOs) by the World Health Organisation in Europe (WHO Eur) [20]. The EPHOs describe the main tasks of public health and can be used as a unifying and guiding basis to monitor and evaluate policies, strategies and actions for reforms and improvement in public health.

The environmental public health workforce was defined as all workers who contribute to the delivery of environmental public health. We made this definition operational as: all those who consider environmental public health as part of their job and who are responsible for providing any of the EPHOs for (on average) ≥ 0.5 h/week. This small number of hours per week was chosen to capture all disciplines and services provided. For example, the work of the health care inspectorate includes promotion of public health and responsible care through effective enforcement of the quality of health services. For environmental public health these services are delivered only 2–3 weeks per year.

As the EPHOs are not yet implemented in public health practice in the Netherlands, we operationalized the EPHOs to environmental public health using existing policy documents, e.g. from the professional organisation of environmental public health physicians, guidelines on the size of the environmental public health workforce and the most recent advice of the Advisory Committee on Medical Manpower planning [Capaciteitsorgaan] on the training inflow of environmental public health physicians [21]. We also involved a group of 5 national environmental public health experts who agreed on the resulting specifications. Based on the documents and the expert opinions, we made some changes to the EPHOs: EPHO ‘advocacy communication and mobilisation for health’ was combined with EPHO ‘health promotion’. We decided to combine these two EPHOs as ‘advocacy communication and mobilisation for health’ according to WHO eur contains improving health literacy and enhancing population’s capacity to access, understand and use information to reduce risk or prevent disease [22]. These kind of activities are part of health promotion in the Netherlands. ‘Regional consultation and support’ was added as EPHO as some local public health services fulfil this specific role for other local public health services. A description of environmental public health and the EPHOs is presented in Table 2.

**Table 2:** Essential public health operations and environmental public health operations in the Netherlands

	Essential public health operations, WHO^a^		Essential environmentalpublic health operations	Examples of daily practice
1	Surveillance of population health and wellbeing	1	Surveillance, evaluation, and analysis of (the determinants of) environmental health and wellbeing	Monitor and register notifications and questions of citizens
				Add questions regarding environmental health to the national health monitor
2	Monitoring and response to health hazards and emergencies	2	Monitoring and response to environmental health hazards and emergencies	Communication of health risks after small incidents
				Follow-up and monitoring of health complaints after a fire containing asbestos
3	Health protection including environmental occupational, food safety and others	3	Health protection, enforce laws and regulations that protect environmental health and ensure safety	Including health in the revised law on intensive farming
4	Health Promotion including action to address social determinants and health inequity	4	Health promotion, including action to address social determinants, health inequity and health literacy	Organise information sessions about health effects of atmospheric pollution
				Campaigning for healthy climates inside buildings and houses
9	Advocacy communication and social mobilisation for health	4	Health promotion, including action to address social determinants of environmental public health, health ine + D20quity and health literacy	
5	Disease prevention, including early detection of illness	5	Disease prevention, diagnosis and investigation of environmental health problems and health hazards	Analyse and follow-up of notifications from citizens
				Active research on healthy enviroments within schools and learning outcomes
6	Assuring governance for health and wellbeing	6	Assuring governance for health, support environmental health public policy	Advise local governments on new housing next to power pylons
				Advise local governments on how to handle asbestos in primary schools
7	Assuring a sufficient and competent public health workforce	7	Assuring a sufficient and competent environmental public health workforce	Supervision of trainees
				Development of a curriculum on environmental public health
8	Assuring sustainable organisational structures and financing	8	Assuring sustainable organisational structures, enforcement of the quality of health services	Participation in quality policy like the Harmonisation Quality Evaluation in the social service sector (HKZ)
				Enforcing quality + E29 of health care (organisation and quality of environmental public health services)
10	Advancing public health research to inform policy and practice	9	Advancing research and development on environmental public health	Conducting scientific research on environmental public health
				Development of new health promotion materials to promote environmental public health
		10	Regional consultation and support	Advising neighbouring environmental public health services

^a^European Action Plan for Strengthening Public Health Capacities and Services

Malta: World Health Organisation, regional office for Europe, 2012

According to Dutch law, formal ethical approval was not required, but we took every effort to effectively inform the respondents and protect their privacy.

### Development of the questionnaire

The questionnaire was developed based on a review of literature, interviews with public health experts and consultation with other researchers and contained 20 items divided into three parts:

1. Eligibility and socio-demographic variables: Is environmental public health part of your job and do you spend more than 0.5 h per week on average on environmental public health tasks? The questionnaire ended if a respondent did not fulfil these criteria. Items on age, gender and educational background (level and discipline, specific training in environmental public health) completed this section.
2. Job characteristics: Type of organisation, job title, and number of years of work experience in the current job.
3. EPHOs: For each separate EPHO, respondents were asked to indicate explicitly if they delivered this operation, and if yes, the average time spent on each of them per week. To facilitate completing this part of the questionnaire examples of daily environmental public health practice were added to each of the EPHOs, see Table 2 Finally, respondents were asked whether they had enough time to perform these operations.

Testing of the practicality of the questionnaire among all employees of a local public health service (n = 217) resulted in some modification of the wording and the order of some items For the present study the adapted version was used. After some adaptations based on a pre-test of this questionnaire among 5 environmental public health workers, the questionnaire took about 10 min to complete.

### Recruitment of participants

Potential respondents were selected in a two-layered recruitment strategy. First we identified all organisations likely to conduct environmental public health tasks and second, within these, we invited all workers considered to be performing environmental public health.

To enhance recruitment across organisations and of as many employees and disciplines substantially involved in environmental public health, we composed two complementary mailing lists. In the first mailing list (core group; n = 182), we included all workers of the departments of environmental public health of the local public health services. Then, we explored who might also be likely performing environmental public health EPHOs outside the department of environmental public health of the local public health services, and included those addresses in the second mailing list (peripheral group; n = 290). For example, in order to recruit respondents involved in EPHOs ‘surveillance’ and ‘health promotion’ we approached all workers from the divisions of epidemiology and health promotion of the local public health services. Similarly, we approached direct network contacts, like employees of the Ministry of Health, Welfare and Sport, environmental public health workers from the National Institute for Public Health and the Environment and departments of public health of two universities in order to recruit workers involved in EPHO ‘advancing public health research’ and EPHO ‘governance for health’.

Both mailing lists were composed in collaboration with environmental public health experts and the national association organisation of all local public health services (GGDGHOR-Netherlands, Table 1). This organisation supports environmental public health practice, policy and research from a national perspective and maintains a good overview of the national environmental public health network.

### Data collection strategy

The survey was performed in March 2013. The invitation to participate in the survey was distributed by e-mail to 472 workers. The invitation emphasized voluntary participation and responses would be confidential. The e-mail contained a link to a secured website where they could complete the electronic questionnaire [23]. In the week after the invitation, two reminders were sent to the non-responders. After two weeks the database was closed, data were downloaded, and the analyses were performed with SPSS 14.0.

### Analysis

#### Feasibility

The feasibility of the measurement strategy was assessed by:

- The complete response rate;
- The number of partial respondents (defined as respondents who started the questionnaire without completing it);
- Remarks added by respondents.

#### Validity checks

External data provided the opportunity to check aspects of the validity of the strategy.

- Existing benchmark data for specific groups: environmental public health physicians and the total capacity of environmental public health workers at the local public health departments:
  The assessment of the capacity of environmental public health physicians in 2010 by the Advisory Committee on Medical Manpower Planning; the total capacity was 14 physicians [21].
  The total capacity of environmental public health workers within local public health departments was enumerated in 2011; the total capacity was 75.5 FTEs.
- Feedback on the results from national experts on environmental public health, as a test of face validity.
  The group of 5 national experts consisted of representatives of the medical environmental public health professional organisations, the national association of all local public health services and two managers of departments of environmental public health from two local public health services. We organised a group session with the experts and after we presented the results we asked the experts to give feedback on:
  whether they recognized the data on the size and composition of the workforce;
  whether the EPHO profile was a reasonable reflection of the actual situation in practice.

#### Size, composition and services provided

Characteristics of the composition of the workforce included, gender, educational level and background, specific training in environmental public health, work setting, job title, and years of work experience in the current job title.

The size of the workforce was calculated in full-time equivalents (FTEs): In the Netherlands, 36 working hours/week constitutes 1 FTE.

For a tentative estimation of the total FTEs of the national environmental public health workforce, we assumed that:

- all non-responders from the peripheral group were not involved in environmental public health for more than 0.5 h/week
- the proportion of environmental public health workers among the respondents of the core group was the same as among the non-responders of the core group environmental public health workers among the non-responders of the core group spent a similar number of working hours on environmental essential public health operations as respondents of the core group. To gain insight into the services provided by the professionals, we assessed which of the EPHOs they provided and for how many hours per week. The distribution of FTEs over the EPHOs was calculated as the sum of all the hours spent per EPHO, divided by 36.

## Results

### Feasibility

The response rate among the local public health services was 100 %. Within the organisations, the response rate of individual workers was 70 % (127/182) in the core group and 28 % (81/290) in the peripheral group. After exclusion of respondents who reported not to be involved in environmental public health or who reported spending ≤ 0.5 h/week (n = 59), and double (n = 26) and partial respondents (n = 14), 129 questionnaires were available for analysis: 112 from the core and 17 from the peripheral group. As the characteristics of both respondent groups were similar for educational background, job titles and involvement on EPHOs, we combined the data from these two groups.

Of all respondents, 26 added remarks to the questionnaire. Of these, 6 indicated that the number of hours spent per EPHO was difficult to estimate and 3 reported not to recognize the EPHOs as a reflection of their daily practice. The other comments were personal additions to the answers.

### Validity checks

Existing benchmark data:

- Our data showed that 28 respondents were physicians, of which 10 had been trained and registered as environmental public health physicians. Data from the Advisory Committee on Medical Manpower Planning showed 14 registered environmental public health physicians in 2010. As all 14 environmental public health physicians were included and invited in the core group, we assumed that the difference was explained by non-response of 4 physicians in our survey, corresponding with the 70 % response rate in the core group.
- Local public health services: The total number of 97 FTEs in our study exceeded the number of 75.5 FTEs from existing reference data. According to the expert group, this could partly be explained because the reference study focused on employees working at the environmental public health department of the local public health services only, whereas our study also included employees from other departments of the local public health services and other organisations.

National experts in the field of environmental public health indicated that the data were recognizable as a reasonable reflection of the actual situation with regard to educational background, employment setting, task differentiation and the provision of EPHOs.

- The experts recognized that the majority of the workforce was trained at university level or higher.
- They also found that the amount of FTEs provided outside the local public health services was plausible. The finding that respondents with a lower educational background spent more time on the EPHOs ‘surveillance’, ‘disease prevention’ and ‘health promotion’, whereas physicians spent more time, e.g., on ‘assuring governance for health’ and ‘advancing research’ was, according to the experts a valid reflection of the situation in daily practice.
- Finally, the distribution pattern of the FTEs over the EPHOs was in line with the expectations of the experts. The results showed more capacity for the EPHOs focusing on daily environmental public health problem-solving, like ‘assuring governance for health’, ‘disease prevention’ and ‘health promotion’, than on the more supportive EPHOs like ‘assuring sustainable organisational structures’ and ‘assuring a competent workforce’. According to the experts this is in line with the actual situation, although the experts had expected more capacity spent on ‘assuring governance for health’.

### Composition of the environmental public health workforce

Of all respondents, the mean age was 46 years (SD 10.2), 64 % was female, 71 % had special training for environmental public health, 59 % had ≥ 5 years working experience in their current job, and 80 % was educated at university level (or higher) with various disciplinary backgrounds (e.g. medicine, nursing, toxicology, biology, and environmental hygiene) (Table 3). Respondents had multiple different job titles. Of the primary job titles 79 % could be classified in 5 main categories (policy advisor, environmental public health physician, environmental public health nurse, environmental public health advisor, and environmental public health emergency expert); the remaining 21 % consisted of 22 different job titles.

**Table 3:** Characteristics of the environmental public health workforce

	Total (n = 129)	
Gender, female N (*%*)	83 (64)	
Age, yrs (*SD*)		46.1 *(±10.2)*
Number of years in current job (%)		
<1 year	6 *(5)*	
1–5 years	47 *(36)*	
6–10 year	42 *(33)*	
> 10 year	34 *(26)*	
Working hours/week hrs (*SD*)		30 *(±7.1)*
Working hours/week environmental public health hrs (SD)		22 *(±11.4)*
Education N (*%*)		
≤Senior secondary vocational education and training	2 (2)	
Professional eduction, applied science	24 (19)	
University level	65 (50)	
Post university level	38 (29)	
Physicians N (%)	28 *(22)*	
Environmental public health physician	10 *(8)*	
General public health physician	10 *(8)*	
Physician	8 *(6)*	
Organizational setting N (%)^a^		
Local public health service	118 (92)	
National	8 (6)	
Municipality	4 (3)	
University	3 (2)	
Other	3 (2)	
Special training for Environmental Public Health n (%)		
Yes	92 (71)	
No	37 (29)	

^a^Multiple work settings per person are possible

Twenty eight Respondents were physician, 10 were trained as environmental public health physician, 10 were trained as public health physician and 8 were physicians ‘other’, of which 6 were in training for environmental public health physician.

Of all respondents, 92 % were working in local public health services, 13 % worked outside the local public health services (e.g. university, Ministry, or research institute), some had multiple work settings, and 45 % were working in environmental public health and in other domains (e.g. infectious disease control).

### Services provided by the environmental public health workforce

**Fig. 1:**
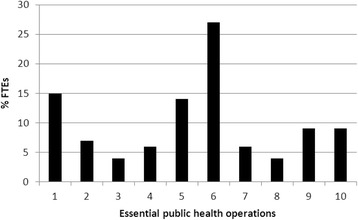
Percentage of full time equivalents per environmental essential public health operation. 1. Surveillance, evaluation, and analysis of (the determinants of) environmental health and wellbeing. 2. Monitoring and response to environmental health hazards and emergencies. 3. Health protection, enforce laws and regulations that protect environmental health and ensure safety. 4. Health promotion, including action to address social determinants, health inequity and health literacy. 5. Disease prevention, diagnosis and investigation of environmental health problems and health hazards. 6. Assuring governance for health, support environmental health public policy. 7. Assuring a sufficient and competent environmental public health workforce. 8. Assuring sustainable organisational structures, enforcement of the quality of health services. 9. Advancing research and development on environmental public health .10. Regional consultation and support

Figure 1 shows the distribution of the capacity across the 10 EPHOs. All EPHOs were provided; most FTEs were spent on the essential operations ‘Surveillances of (the determinants of) population (environmental) health and wellbeing’ and ‘assuring governance for health’ and the least FTEs were spent on the EPHOs ‘health protection’ and ‘assuring sustainable organisational structures’. See Table 4 for the specific percentages and the average hours per week spent per EPHO.

**Table 4:** Specification of percentage FTE-, time of physicians and nurses and, hours per week per essential environmental public health operation

		% Total FTE	Average hrs/week (SD)	% Time physician	% Time nurse
1	Surveillances, evaluation, and analysis of (the determinants of) environmental health and wellbeing	15	4.4 (5.1)	8.8	22
2	Monitoring and response to environmental health hazards and emergencies	7	1.9 (2,7)	8.4	3.3
3	Health protection, enforce laws and regulations that protect environmental health and ensure safety	4	1.9 (2.5)	5.6	1.5
4	Health promotion, including action to address social determinants, health inequity and health literacy	6	1.8 (1.9)	4.8	10.2
5	Disease prevention, diagnosis and investigation of environmental health problems and health hazards	14	4.6 (5.7)	8.9	34.3
6	Assuring governance for health, support environmental health public policy	27	8.4 (4.5)	26.2	9,6
7	Assuring a sufficient and competent environmental public health workforce	6	1.8 (1.9)	9.6	5.9
8	Assuring sustainable organisational structures, enforcement of the quality of health services	4	1.3 (1.3)	2.8	8.9
9	Advancing research and development on environmental public health	9	3.5 (5.3)	11.4	2.4
10	Regional consultation and support	9	3.9 (5.0)	13.5	1.8

Compared to environmental public health physicians, environmental public health nurses were less often involved in performing the EPHOs, ‘Advancing research’ and ‘regional consultation and support’ (Table 4).

### Total size of the environmental public health workforce

The total capacity of the environmental public health workforce was estimated at 110 FTEs (range 79–152 FTEs) or 0.66 FTE per 100,000 inhabitants. Of the total FTEs, 97 % (96 FTEs) was provided through the local public health services and 13 FTEs through other organizations.

## Discussion

We developed and applied a strategy to enumerate the size and composition of the general public health workforce and the services provided, on the basis of actual involvement in EPHOs. Quantitative estimates of self-reported individual respondents’ actual involvement in EPHOs collected through an online survey proved to be a useable source for national estimates of the environmental public health workforce. The questionnaire was well accepted and available benchmark data on physicians and the total number of environmental public health professionals working at local public health services supported the validity of the results emerging from the present study. In the Netherlands, the total size of the environmental public health workforce was estimated at 0.66/100,000 inhabitants. The EPHOs were provided through different organizations and performed by an experienced, highly educated and multidisciplinary workforce.

Our strategy started by specifying the EPHOs to environmental public health, followed by identifying the organizations and individuals considered to be involved in delivery of environmental public health. Because this resulted in inclusion of workers outside local public health service and from other disciplines than environmental public health as well, the estimation of the total capacity for environmental public health was—as expected—higher than previously estimated on the basis of registries. Furthermore the reference data were from 2011, and might explain a difference in workforce size as well.

In addition, our strategy resulted not only in data on the number of workers, but also in additional data on the composition of the workforce, educational background and the provision of EPHOs. Our study clearly demonstrates the multi-disciplinarity of the workforce in terms of educational degree as well as in background. Only 22 % of the respondents was physician and the remainder had various educational backgrounds. The current health human resource planning model used in the Netherlands by the Advisory Committee on Medical Manpower planning [Capaciteitsorgaan] is a mono-professional model to estimate the training inflow of physicians, including environmental public health physicians. Taking our results into account, implies that central workforce planning is only targeting 22 % of the total workforce [21]. To address the whole workforce health human resource planning should be integrated.

Our study also revealed data on the EPHOs provided, by whom and for how many hours. A next step to support workforce planning would be to examine whether corresponding competencies are sufficiently addressed in the different curricula [24, 25].

Whereas we demonstrated the feasibility of this new strategy for enumerating public health workforce for one specific example—the environmental public health workforce—we believe that this strategy can be applied to other public health sectors as well. The universal applicability is the result of the fact that this strategy has been based on EPHOs. Our approach is therefore also applicable to assess the capacity of the total public health workforce in the Netherlands or abroad, as WHO’s EPHOs were considered valid for all WHO Eur countries [20].

Although the feasibility of our measurement strategy was demonstrated and resulted in more insight in the size and composition of the environmental public health workforce than was available before from registry data, this first application brought some limitations to light that need to be addressed in further development. These include the selection of the environmental public health organisations; workers who were not used to classify their work according to the EPHOs; and the response rates.

Our estimation of the total capacity was dependent on proper identification of organizations harbouring people belonging to the environmental public health workforce. We explored per EPHO who or which organizations potentially perform that service, and decided to compose a core group and a peripheral group. The core group was identified through organisations who were likely to be involved in delivering environmental public health EPHOs and the peripheral group of those who might be involved in environmental public health. It could be that our selection was incomplete and in that case the total available capacity estimated in this study may be an underestimation of the real capacity. However, overestimation is also possible.

Workers were asked to classify there daily tasks according to the EPHOs and were asked to estimate the average time they spent on each of EPHOs per week. As the EPHOs were only recently introduced in Europe, workers are not yet familiar with linking their daily work to the EPHOs. This may have resulted in misclassification. For example some workers might have classified a specific task to a certain EPHO whereas it should have been classified to another EPHO. To prevent misclassification and to shape workers’ mental image of the tasks to be assessed, we added examples from daily practice to each of the EPHOs. As the group of environmental health experts recognized the overall results of the study as being in line with the provision of EPHOs in daily practice, we assume workers were able to classify there daily tasks to each of the EPHOs properly. However, further validation of the correctness of the classification and quantitative estimation of hours spent is required in further studies.

## Conclusion

We developed and applied a novel strategy to enumerate the public health workforce based on assessment of individual workers’ involvement in EPHOs. We identified relevant organisations and individuals on the basis of EPHOs and obtained information from those individuals by using online questionnaires. Critical factors include the selection and recruitment of eligible organisation and the response rate. When existing professional registries are incomplete or do not exist, this strategy may provide a start to enumerate the quantity and quality of the public health within or across countries.
